# Sexual Function in Patients Suffering from Sacrococcygeal Pilonidal Sinus Disease

**DOI:** 10.7759/cureus.7159

**Published:** 2020-03-02

**Authors:** Akke Pronk, Lotte Kastelijns, Niels Smakman, Edgar Furnee

**Affiliations:** 1 Surgery, Diakonessenhuis, Utrecht, NLD; 2 General Medicine, Royal Dutch Army, Utrecht, NLD; 3 Surgery, The University Medical Center Groningen, Groningen, NLD

**Keywords:** sacrococcygeal pilonidal sinus disease, sexual function, prospective cohort study, quality of life

## Abstract

Introduction

Sexual function is one of the aspects upon which quality of life (QoL) is based. Although previous studies have evaluated the influence of sacrococcygeal pilonidal sinus disease (SPSD) on QoL, no data are available on the influence of SPSD on sexual function in a highly active sexual population based on the age range. The aim of this prospective study was to evaluate whether SPSD has a negative impact on sexual function and whether this is influenced by the surgical treatment of SPSD.

Methods

Sexual function was pre- and postoperatively assessed by the Sexual Self-Consciousness Scale (SSCS; score range 0-48), subdivided into the sexual embarrassment (SE; score range 0-24) and sexual self-focus subscale (SFF; score range 0-24). The higher the score, the higher is the sexual dysfunction. Patients were also asked whether SPSD influenced their sexual functioning.

Results

A total of 88 male patients who underwent surgical treatment for SPSD were included in the study. The mean (±SD) preoperative SSCS score was 14.5±9.1 and 13.9±8.4 two weeks postoperatively (p=0.394). Six and twelve weeks after surgery, there was a significant reduction to 12.2±9.0 (p=0.002) and 12.3±8.8 (p=0.013), respectively. SE decreased from 5.5±5.1 preoperatively to 5.1±4.6 (p=0.258), 4.2±4.7 (p=0.004) and 4.0±4.6 (p=0.013) two, six, and twelve weeks after surgery. For SFF, there was a decrease from 9.0±5.0 to 8.9±4.9 (p=0.717), 7.8±5.2 (p=0.004) and 8.2±5.3 (p=0.168), respectively. Preoperatively, 70% of the patients totally or partially disagreed that SPSD influenced their sexual functioning, and this increased to 80% of the patients 12 weeks after surgery.

Conclusion

This prospective study showed a significant decrease in sexual dysfunction, both six and twelve weeks after surgery, compared to preoperatively in patients suffering from SPSD.

## Introduction

Sacrococcygeal pilonidal sinus disease (SPSD) is an acquired disorder of the natal cleft. SPSD affects around 0.7% of the population and is twice more common in men. SPSD is most commonly found between the age of 15 and 30 years, affecting women at a younger age than men [1, 2]. SPSD might present either as an acute abscess or chronic disease. In the acute stage, patients mainly complain of pain, while patients with chronic SPSD often present with intermittent discharge, irritation, itching, or a burning sensation at the natal cleft [1, 3]. Subjective perception of symptoms and the influence of the symptoms on quality of life (QoL) is increasingly taken into account in patients with benign diseases. In SPSD, symptoms have proven to have a negative impact on the quality of life [4].

Sexual function is one of the aspects upon which quality of life is based. Therefore, impairment of sexual function can have a substantial negative impact on QoL. Previous studies have shown a significant increase in the prevalence of sexual dysfunction in patients with chronic pain as well as in patients suffering from chronic diseases such as Parkinson’s disease, epilepsy, chronic renal failure, multiple sclerosis, and dermatological diseases [5]. Although several studies have evaluated the effect of SPSD on general quality of life, there are currently no data available on the relationship between sexual (dys)function and SPSD. In addition, specific data concerning the change of sexual function after treatment of SPSD have never been investigated. However, since patients affected by SPSD are within the age range of a sexually active population, data on sexual function is highly relevant [6].

This prospective study aimed to evaluate whether SPSD has a negative impact on sexual function and whether this is influenced by the surgical treatment of SPSD.

## Materials and methods

Patients with SPSD were identified from a randomized controlled trial (RCT) comparing two different treatment modalities for primary SPSD, including phenolisation of the sinus tract and radical excision [7]. Patients over the age of 18 with symptoms due to primary chronic SPSD were included in the RCT. Exclusion criteria were no or minimal symptoms related to SPSD, suspicion of an extensive subcutaneous network of sinus tracts as this is a contra-indication for phenolisation, presence of an abscess, or previous surgery related to SPSD. Since only a few female patients were included in the RCT due to the lower incidence of SPSD in the female population, only male patients were included in the current analysis to obtain the results on sexual function in a more homogenous group of patients. Since the aim of this study was to evaluate sexual function in patients with SPSD, sexual function was analyzed independently of the type of surgery.

Data collection 

Preoperatively, baseline characteristics were prospectively collected, including age, body mass index, smoking, relationship status and family history of SPSD. Information regarding sexual function was preoperatively obtained and in addition, two, six and twelve weeks postoperatively by the Sexual Self-Consciousness Scale (SSCS) [8]. The SSCS is a 12-items questionnaire, assessing sexual function based on sexual self-consciousness. The questions included in the SSCS are shown in Table 1. The SSCS can be subdivided into two six-items subscales, including Sexual Embarrassment (SE) subscale and Sexual Self-Focus (SSF) subscale. All items were answered on a five-point scale (score range: 0-4), resulting in a minimum score of zero and the maximum score of 24 points per subscale, and a cumulative maximum score of 48 points on the complete SSCS. Increasing test scores correlate with more sexual self-consciousness and, therefore higher sexual dysfunction. In addition, participants were also asked to indicate to what extent they agree with the statement "Pilonidal sinus disease influences my sexual functioning" with available answers "totally disagree", "partially disagree", "I don’t know", "partially agree" or "totally agree". 

**Table 1:** Sexual Self-consciousness Scale questionnaire Score range 0-4 per item: 0 - strongly disagree; 1 - disagree a little; 2 - neither agree or disagree; 3 - agree a little; 4 - strongly agree

Statements in the scales
Sexual embarrassment subscale
It takes quite some time for me to overcome my shyness in sexual situations
I quickly feel embarrassed in sexual situations
I feel uncomfortable in sexual situations
I find it difficult to sexually let myself go in front of the other person
When I see myself during sex, I am irritatingly aware of myself
I continuously feel being observed by the other person during sex
Sexual self-focus subscale
I am aware during sex of the impression I make on the other person
I pay much attention to my sexual thoughts and feelings
I often wonder during sex what the other person thinks of me
I am preoccupied by the way I behave sexually
During sex, I pay much attention to what happens inside my body
I often imagine how I behave during sex

Quality of life was pre- and postoperatively evaluated by the Short Form 36 (SF-36), consisting of 36 questions comprising nine different domains of quality of life: physical functioning, physical role limitation, emotional role limitation, bodily pain, vitality, mental health, social functioning, general health and health change [9]. For every domain, a score between 0 and 100 can be obtained; the higher the score, the better is quality of life.

Additionally, wound closure, defined as complete epithelization of the skin at the natal cleft and fluid discharge from the surgical wound, scored on a six-point scale from 0 (no complaints) to 5 (daily complaints), were postoperatively assessed.

Statistical analysis

Data were analyzed using SPSS for Windows version 23.0 (IBM Inc., Armonk, USA). Categorical data were presented as frequencies with percentages. Continuous values were expressed as mean ± standard deviation (SD). The paired samples t-test and Wilcoxon signed-rank test were used for statistical analysis of continuous pre- and postoperative values. Statistical analysis of values between groups was performed using Mann-Whitney U tests. All tests were two-tailed and were considered statistically significant with p<0.05.

To identify independent predictors of SSCS-scores twelve weeks after surgery, univariate regression analysis was performed by linear regression analysis for correlations between individual variables and SSCS scores. Variables that were analyzed included age, body mass index (BMI), smoking, relationship status, family history of SPSD, number of preoperative sinus pits and postoperative bodily pain, fluid discharge and wound epithelialization at twelve weeks. Variables with p ≤ 0.200 in univariate analysis were all together entered into a multivariate regression model. Variables with p³0.100 in multivariate analysis were excluded until only significant independent variables left (p<0.100). 

## Results

**Figure 1:**
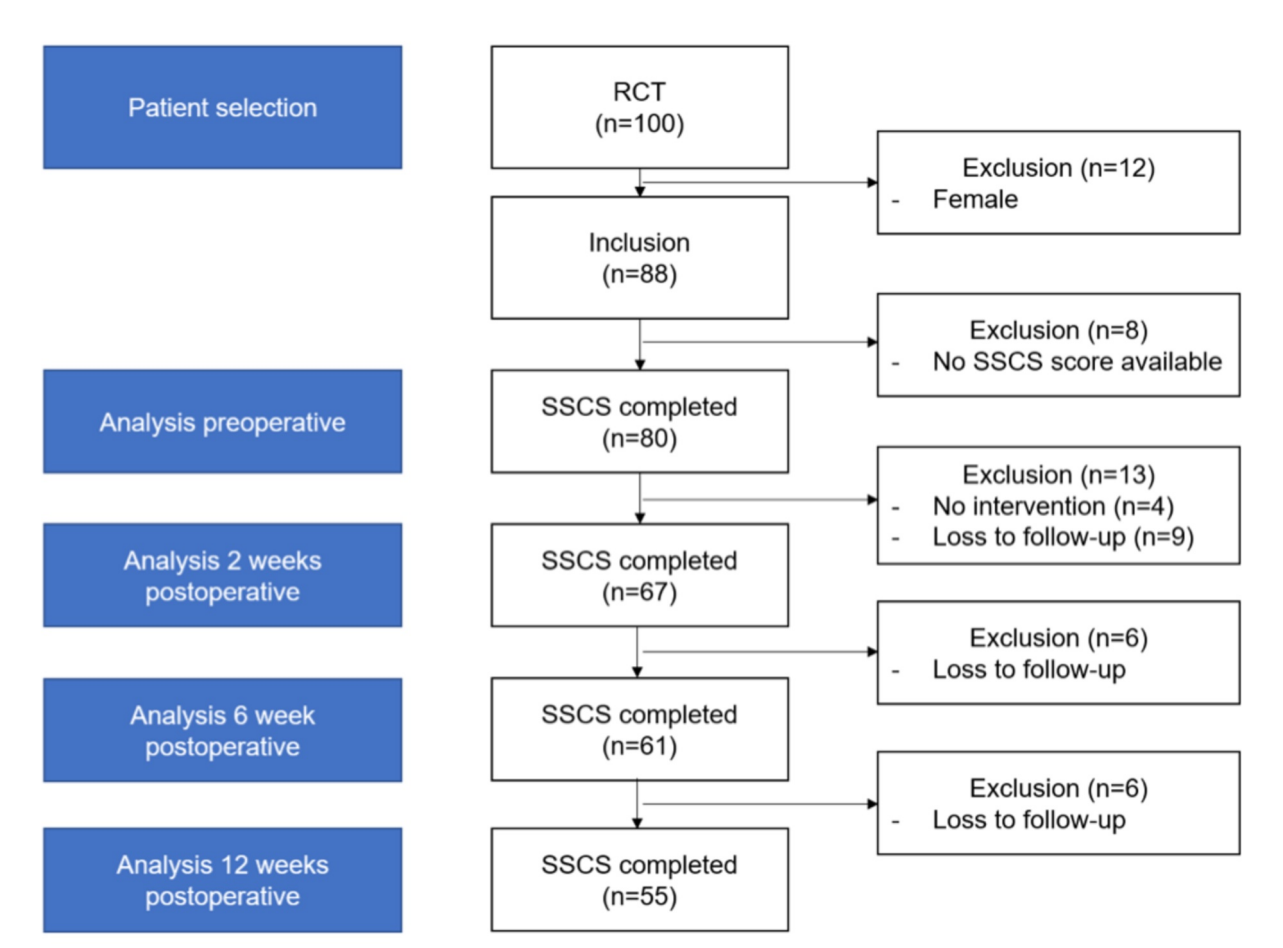
Study flowchart RCT - randomized control trail; SSCS - Sexual Self-Consciousness Scale

Between September 2013 and September 2017, a total of 100 patients were included in the randomized trial. Since twelve of these patients were females, 88 male patients were included in the current analysis. The baseline characteristics and postoperative fluid discharge and wound epithelialization rate of the included patients are shown in Table 2. The flow chart indicating the number of patients who completed the SSCS questionnaire preoperatively and two, six and twelve weeks after surgery is shown in Figure 1.

**Table 2:** Baseline characteristics Values are reported as mean ± standard deviation (SD), unless otherwise stated. * Score range: 0 (no complaints) - 5 (daily complaints) SPSD - sacrococcygeal pilonidal sinus disease

	Total (n= 88)
Age (years)	27.6 ( 8.5)
Body mass index (kg/m^2^)	24.9 (3.6)
Smoking (%)	30 (34.1)
Relationship (%)	51 (58.0)
Positive family history of SPSD (%)	20 (22.7)
First-degree family member	15 (17.0)
Number of preoperative sinus openings	3.56 (1.96)
Type of surgery	
Phenolisation (%)	51.1
Radical excision (%)	48.9
Fluid discharge^*^	
Preoperative	1.8 (1.2)
2 weeks postoperative	1.3 (1.2)
6 weeks postoperative	0.6 (0.9)
12 weeks postoperative	0.5 (1.1)
Complete wound epithelialization	
2 weeks postoperative (%)	11 (12.5)
6 weeks postoperative (%)	40 (45.5)
12 weeks postoperative (%)	52 (69.1)

Sexual function

Two weeks after surgery, the SSCS score was not significantly different compared to preoperatively (Table 3). However, six and twelve weeks after surgery, the SSCS score was significantly lower compared to preoperatively, indicating a better sexual function. The SE subscale showed the same trend as the total SSCS score with a significant decrease from 5.5±5.1 preoperatively to 4.2±4.7 six weeks and 4.0±4.6 twelve weeks after surgery. The SSF subscale also showed a significant reduction six weeks after surgery; however, twelve weeks after surgery there was no significant difference in comparison with the preoperative score. At all three postoperative follow-up points, there was no significant difference in the total SSCS score between patients with and without complete wound epithelialization (Table 3).

**Table 3:** Pre- and postoperative SSCS Values are reported as mean ± SD, unless otherwise stated. SSCS - Sexual Self-consciousness Scores; SPSD - sacrococcygeal pilonidal sinus disease; NA, not applicable Increasing scores on the SSCS indicates higher sexual self-consciousness and consequently more sexual dysfunction. *compared to preoperative value; †comparing SSCS score for complete vs. no complete wound epithelialization.

	Preoperative (n= 80)	Two weeks postoperative (n= 67)	p-value*	Six weeks postoperative (n= 61)	p-value*	Twelve weeks postoperative (n= 55)	p-value*
SSCS (range 0-48)	14.5 (9.1)	14.0 (8.3)	0.394	12.1 (8.9)	0.002	12.3 (8.8)	0.013
Sexual embarrassment subscale (range 0-24)	5.5 (5.1)	5.1 (4.6)	0.258	4.2 (4.7)	0.004	4.0 (4.6)	0.003
Sexual self-focus subscale (range 0-24)	9.0 (5.0)	8.9 (4.9)	0.717	7.8 (5.2)	0.004	8.2 (5.3)	0.168
Total SSCS score in patients with							
Complete wound epithelialization	NA	14.7 (8.2)	0.709^†^	12.0 (9.3)	0.839^†^	11.7 (8.2)	0.768^†^
No complete wound epithelialization	NA	13.7 (8.4)		12.4 (8.9)		13.4 (10.6)	

**Figure 2:**
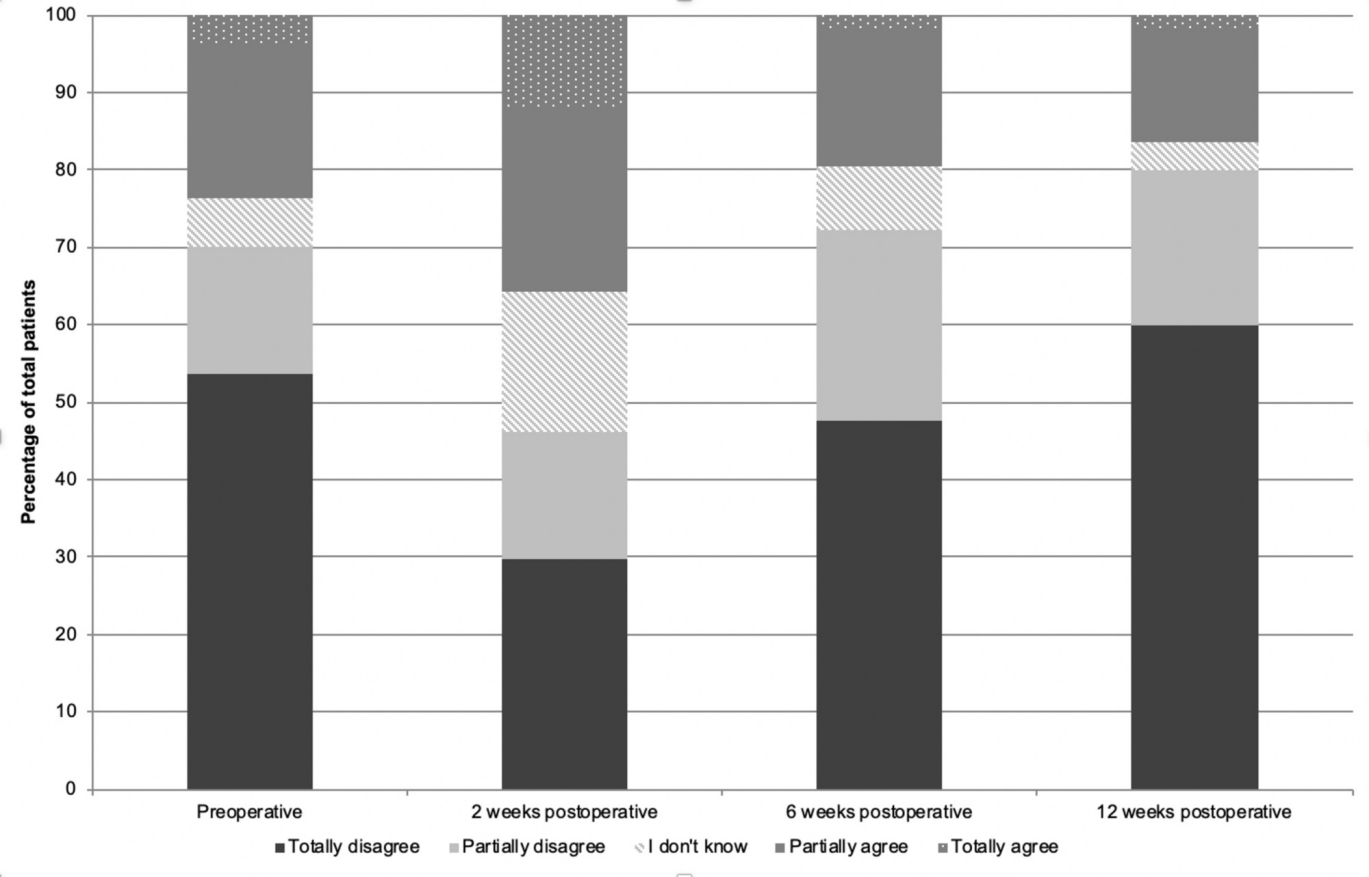
(Dis)agreement with statement "Sinus pilonidal disease influences my sexual functioning"

Preoperatively, 56 patients (70.1%) indicated that their SPSD did not or only partially influenced their sexual functioning (Figure 2). However, two weeks after surgery, patients significantly more often agreed with the statement "Pilonidal sinus disease influences my sexual functioning" compared to preoperatively (totally disagreed 53.8% and totally agreed 3.8% preoperatively vs. totally disagreed 29.9% and totally agreed 11.9% two weeks after surgery, p=0.002). Compared to preoperatively, six and twelve weeks after surgery 2.0% and 9.9% more patients totally or partially disagreed that SPSD influenced their sexual functioning (p=0.861 and p= 0.129, respectively).

Quality of life 

**Figure 3:**
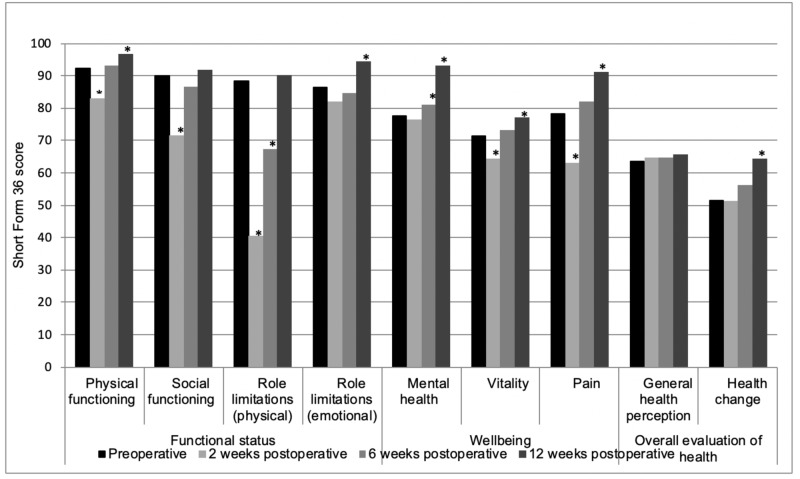
Quality of life (Short Form 36) * p<0.05 compared to preoperatively

The SF-36 domains "physical and social functioning", "physical role limitations", "vitality" and "pain" all decreased significantly two weeks after surgery (Figure 3). However, twelve weeks after surgery, the scores on these domains came back to preoperative levels ("social functioning" and "physical role limitations") or were significantly improved ("physical functioning", "vitality" and "pain"). Three other domains, including "emotional role limitations", "mental health" and "health change" were also significantly improved twelve weeks after surgery compared to preoperatively, without a drop two or six weeks after surgery. The domain "general health perception" did not significantly differ at any moment after surgery compared to preoperatively. 

Uni- and multivariate analysis of SSCS score twelve weeks after surgery

The results of uni- and multivariate analysis for the SSCS score twelve weeks after surgery are shown in Table 4. A total of four variables, including age, relationship status, pain and fluid discharge, found in the univariate analysis were entered into the multivariate analysis. Finally, two variables were identified as significant predictors of the SSCS score twelve weeks after surgery; patients with a relationship and pain independently predicted a higher SSCS score, i.e. more sexual dysfunction. 

**Table 4:** Uni- and multivariate analysis of SSCS 12 weeks after surgery CI - confidence interval for coefficient; SSCS - Sexual Self-Conscious Scale; BMI - body mass index; ns - not significant

Variable	Univariate analysis				Multivariate analysis			
	Coefficient	95% CI		p-value	Coefficient	95% CI		p-value
Age	-0.263	-0.512	-0.014	0.039				ns
BMI	0.347	-0.276	0.970	0.268				
Family history	-0.718	-6.249	4.813	0.795				
Smoking	0.279	-4.699	5.256	0.911				
Relationship status	-5.826	-10.881	-0.772	0.025	-4.068	-8.897	0.760	0.097
Preoperative number of sinus pits	0.200	-1.069	1.468	0.753				
Pain	-0.227	-0.367	-0.087	0.002	-0.201	-0.341	-0.061	0.006
Wound epithelialization	0.001	-0.006	0.009	0.758				
Fluid discharge score	2.432	0.310	4.553	0.025				ns

## Discussion

This is, to our knowledge, the first prospective study evaluating the influence of SPSD on sexual functioning. The results of this study showed that the treatment of SPSD improved sexual function six and twelve weeks after surgery. A relationship and pain were independent predictors of more sexual dysfunction twelve weeks after surgery. In addition, although there was some decrease two weeks after surgery, quality of life also significantly improved twelve weeks postoperatively on most dimensions. 

Although sexual function improved six and twelve weeks after surgery, there was initially two weeks postoperatively, no influence on sexual function. However, we would have expected an increase in the SSCS score two weeks after surgery due to wound pain, fluid discharge, and a wound at the natal cleft. This finding, the absence of a reduction in the SSCS score two weeks after surgery, supports that symptoms related to SPSD do have the same impact on sexual function as complaints due to surgery for SPSD. On the contrary, compared to preoperatively, significantly more patients indicated that SPSD influenced their sexual function two weeks after surgery. This implies that the surgical wound has a higher impact on sexual function than symptoms related to SPSD. So, it is unclear from our data whether surgery for SPSD has a negative impact on sexual functioning during the first two postoperative weeks. 

It has previously been reported that pain and chronic diseases have a negative impact on sexual function, while sexual activity has been associated with health benefits and longevity [5, 6]. Therefore, it is surprising that no data were available on sexual function in patients with SPSD so far. Some studies on sexual function with regard to perianal diseases that are located around the same body area like SPSD have been published, although data on this subject is also limited. Riss et al. investigated sexual function in patients after surgical treatment for perianal fistulas in Crohn’s disease [10]. The authors concluded that postoperative sexual function, measured by the International Index of Erectile Functioning (IIEF), was not different compared to the general population. On the contrary, Lin et al. found a significantly higher incidence of sexual dysfunction in women after surgery for hemorrhoids [11]. Since data on sexual functioning in patients with disorders close to the urogenital area, i.e., the anal region, are scarce and also inconsistent, a plea for additional studies focussing on this subject is justified.

The SSCS questionnaire is divided into two subscales; the Sexual Embarrassment (SE) subscale representing a feeling of inhibition and discomfort in sexual situations and the Sexual Self-Focus (SSF) subscale representing self-consciousness behavior without affective connotation [8]. The current study showed a significant decrease in the SE subscale six and twelve weeks after surgery compared to preoperatively, while the SSF subscale only showed a significant reduction six weeks after surgery. Since the final SSF subscale score twelve weeks after surgery did not show a difference compared to preoperatively and the SE subscale score did, SPSD might have more impact on sexual embarrassment. This might be explained by the fact that the SSF subscale is about "self-focus" which might be less influenced by a physical disorder than sexual embarrassment. 

Preoperative QoL scores measured by the SF-36 health survey questionnaire were, compared to the normal healthy male population as reported by Van der Zee et al., below average on the domains "social functioning", "emotional role limitations", "mental health", "pain", "general health perception" and "health change" [9]. In more than half of the domains, QoL reduced two weeks after surgery, probably due to the consequences of surgery, including pain, fluid discharge, and a wound at the natal cleft. However, in six of the nine domains of the SF-36, values significantly improved twelve weeks after surgery and were, compared to the normal healthy population, at least equal on all domains, except for "general health perception". Since Jenkinson et al. showed decreased SF-36 scores for patients with a long-lasting illness, the lower scores 12 weeks after surgery in "general health perception", compared to healthy male adults as reported by Van der Zee et al., could probably be explained by the history of the chronic illness SPSD [9, 12].

This prospective study has some limitations that need to be considered. First, the loss of follow-up was relatively high. Despite reminding phone calls, patients failed to return the questionnaire. This might be due to the subject of the questionnaire as patients feel embarrassed to complete sexual-related questions. In addition, some patients only completed the quality of life questionnaire and refused to complete the SSCS. This has contributed to the relatively high loss to follow-up. Second, the follow-up period is relatively short. However, even after twelve weeks of follow-up, a significant decrease in the SSCS score was found. Since about 70% of patients had complete wound healing after twelve weeks of follow-up, longer follow-up is finally expected to lead to complete wound healing in all patients and this might result in an even higher improvement in sexual function. However, a significant difference in SSCS scores between patients with or without complete wound healing was not detected during any of the postoperative follow-up points. So, a further decrease in the SCSS score, in the long run, is speculative. Third, data on the SSCS in the normal healthy population are lacking, so we were not able to compare patients suffering from SPSD at baseline with healthy individuals. In addition, the minimal clinically important change of the SCSS score is also unknown. For that reason, we also obtained from all patients to what extent they agreed with the statement "Pilonidal sinus disease influences my sexual functioning". Since there was an increase from preoperatively to 12 weeks after surgery of 10% of patients who totally or partially disagreed, we considered the significant increase of the SCSS score as clinically relevant. Finally, female patients were excluded from the analysis, so no data on sexual function are available in the female population. This was decided as only 12% of the included patients were women, and excluding them resulted in a more homogeneous study population with only male patients. However, future research should also focus on sexual (dys)function in female patients suffering from SPSD.

## Conclusions

In conclusion, this is, as far as we know, the first study evaluating sexual function after surgery in patients with SPSD, including a patient population in a highly active sexual age range. This prospective study showed a significant decrease in sexual dysfunction six as well as twelve weeks after surgery for SPSD. The significant improvement of sexual function as shown in this study supports surgical treatment in the case of symptomatic SPSD and contributes to the preoperative counselling of patients facing surgery for SPSD. 
